# Guillain-Barré Syndrome After Primary Cytomegalovirus Infection in a Patient With a Heart Transplant

**DOI:** 10.1016/j.jaccas.2020.12.037

**Published:** 2021-03-17

**Authors:** Peter Ting, Anton Camaj, Solomon Bienstock, Alex Choy, Sumeet S. Mitter, Maya Barghash, Donna Mancini

**Affiliations:** a The Zena and Michael A. Wiener Cardiovascular Institute, Mount Sinai Hospital, New York, New York, USA; b Department of Population Health Science and Policy, Icahn School of Medicine at Mount Sinai, New York, New York, USA

**Keywords:** cytomegalovirus, Guillain-Barré Syndrome, heart transplantation, CMV, cytomegalovirus, GBS, Guillain-Barré Syndrome

## Abstract

A 56-year-old man underwent cardiac transplantation in April 2018. His post-operative course was uncomplicated and he had normal allograft function. On December 2019 he was admitted for fever and diarrhea and was found to have cytomegalovirus infection. A few weeks later, he presented with Guillain-Barré Syndrome. (**Level of Difficulty: Advanced.**)

## Graphical abstract

**Figure:**
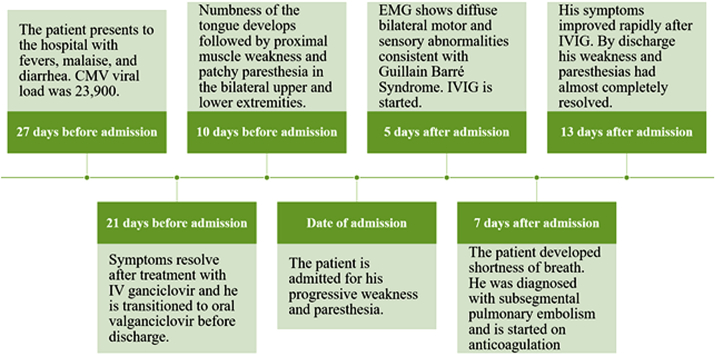

## History of presentation

In January 2020, a 56 year-old-man presented with 10 days of ascending sensory loss and proximal muscle weakness. Physical examination was notable for sensory loss in the lower extremities to just below the knees, in the hands and forearms, and on the anterior tongue. He had weakness of the deltoids, biceps, and intrinsic muscles of the hand, as well as weakness with hip flexion and dorsiflexion of the feet. He had a neuropathic gait. The remainder of his examination, including the cardiopulmonary examination, was unremarkable.

**Learning Objectives**

• To recognize CMV infection as a complication of heart transplantation.
• To recognize the association between CMV infection and GBS.
• To review the presentation and treatment of GBS.

## Patient Medical History

**Figure 1:**
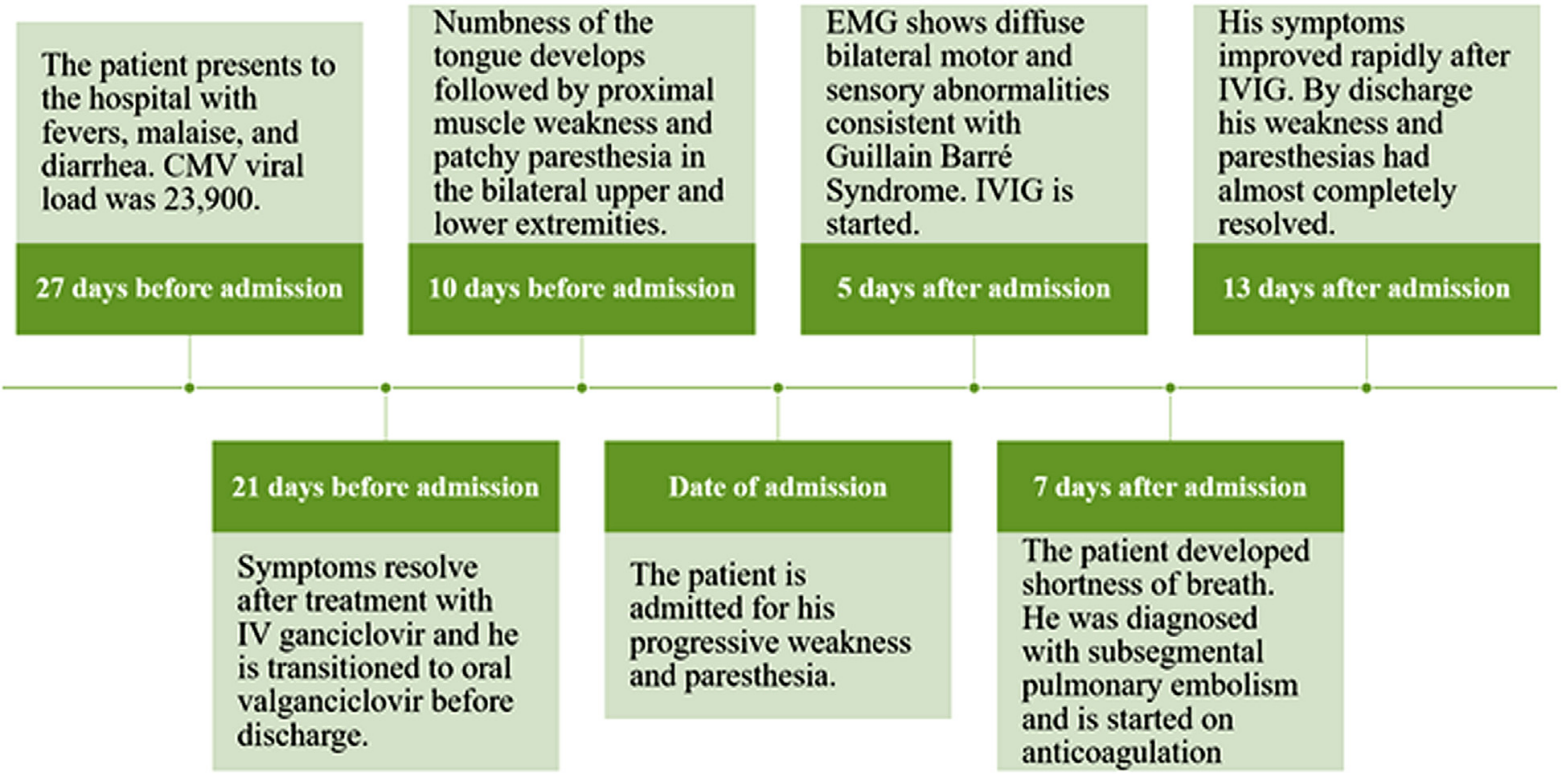
Timeline of the Events Leading to the Patient’s Diagnosis of Guillain-Barré Syndrome, His Treatment With IVIG, and Resolution of Symptoms CMV = cytomegalovirus; IV = intravenous; IVIG = intravenous immunoglobulin.

The patient had a history of end-stage heart failure due to ischemic cardiomyopathy for which he underwent left ventricular assist device implantation as a bridge to cardiac transplantation in April 2018. Both he and the donor were seronegative for cytomegalovirus (CMV). He had normal allograft function without allograft rejection. He was on tacrolimus and mycophenolate mofetil for maintenance immunosuppression. In December 2019, he was hospitalized for CMV infection manifesting as fever and diarrhea. His CMV DNA polymerase chain reaction (PCR) was 23,900 copies/ml, and he was treated with intravenous ganciclovir for 6 days and then transitioned to oral valganciclovir. By discharge, fever and diarrhea had resolved. On follow-up, CMV DNA was undetectable. Three weeks after discharge he presented with progressive muscle and sensory loss (see Figure 1 for timeline).

## Investigations

Laboratory investigations revealed a normal basic metabolic panel and complete blood count. His liver function tests were improving relative to his prior admission.

Creatine kinase was normal. Thyroid-stimulating hormone was 0.009 IU/ml (normal 0.4 to 4.2 IU/ml), free T4 was 1.58 ng/dl (normal 0.8 to 1.5 ng/dl), and free T3 was 6.56 pg/ml (normal 2.5 to 3.9 pg/ml). Thyroglobulin, thyroid peroxidase, and thyroid stimulating antibodies were absent. Thiamine, vitamin B12, serum protein electrophoresis, immunofixation electrophoresis, and copper levels were normal. Human immunodeficiency virus test, rapid plasma reagin, CMV PCR, and Epstein Barr virus PCR were negative. Tacrolimus level was 8.9 ng/ml.

C-reactive protein was 14.4 mg/l (normal 0 to 5 mg/l), and an antinuclear antibodies titer was positive at 1:320 in a speckled pattern. Erythrocyte sedimentation rate was 40 mm/h.

Antibodies to MAG, SGPG, Purkinje Cell/Neuronal Nuclear, Asialo-GM1, GD1A, and GQ1B were negative. GD1b antibodies were elevated to 74 IV (negative <50).

Magnetic resonance imaging of the entire spine showed possible enhancement of the cauda equina nerve roots at the L5-S1 level. Nerve conduction studies were suggestive of early to subacute Guillain-Barré Syndrome (GBS).

## Management

The patient was given 3 days of intravenous immunoglobulin (IVIG), 30 g/day, and the valganciclovir was discontinued given the concern for valganciclovir toxicity. His hyperthyroidism was treated with methimazole.

## Follow-Up

Shortly after the initiation of IVIG therapy, he developed shortness of breath. Right heart catheterization showed borderline high filling pressures but a normal cardiac index. He was empirically treated for rejection with furosemide and intravenous steroids, which were tapered when biopsy was consistent with 1R allograft rejection. Computed tomography angiography showed a small subsegmental PE and he was begun on anticoagulation.

His strength and paresthesia improved following IVIG therapy. On discharge he had mild residual weakness and his peripheral had almost resolved.

## Discussion

Immunosuppressive therapy used to prevent allograft rejection predisposes heart transplant recipients to more infections. A common post-transplant infection is CMV. Here, we report a case of GBS following an episode of primary CMV infection in a patient 21 months post-cardiac transplantation.

The differential diagnosis in this patient included drug toxicity, hyperthyroidism, and GBS. Valganciclovir toxicity and GBS can both cause peripheral neuropathy. However, muscle weakness is not typically associated with valganciclovir peripheral neuropathy, whereas it is a key feature of GBS (1). Rarely, hyperthyroidism can cause neuropathies such as Basedow’s paraplegia though sensory symptoms are usually minimal or absent (2). Furthermore, the temporal relationship of CMV infection and symptom onset is strongly suggestive of GBS. Electromyography and nerve conduction studies confirmed the diagnosis of GBS.

GBS is an autoimmune disease, often occurring after infections such as campylobacter, CMV, Zika, and HIV. It most commonly affects women under the age of 30 years (3). GBS is thought to occur when an infectious organism stimulates the production of an antigen that cross-reacts with epitopes on the peripheral nerves (4). It is typically a progressive, symmetrical muscle weakness beginning in the legs accompanied by paresthesia, although it can also present with arm, facial, or bulbar involvement. Symptoms typically progress over 4 weeks (5). CMV-associated GBS often presents with severe sensory loss and cranial nerve involvement as was the case for our patient (6).

Complications of GBS include neuromuscular respiratory failure, autonomic dysfunction, and long-term neurological disability. Frequent monitoring of the negative inspiratory force and vital capacity is necessary in patients, as 15% to 30% of patients will require ventilatory support for respiratory failure. Autonomic dysfunction can cause tachycardia, fluctuations in blood pressure, arrhythmias, urinary retention, and ileus. At 1 year, approximately 60% of patients will have full recovery of motor strength. GBS can also affect the myocardium leading to myocarditis and subsequent heart failure (5).

Only 6 prior cases of GBS due to CMV infection after heart transplantation have been described (Table 1). Including our case, all were middle-aged men. All except 1 occurred within 2 years of cardiac transplantations. Our patient is the first where the only treatment used was IVIG.

**Table 1:** Clinical Characteristics of 7 Cases of GBS After CMV

First Author (Ref. #)	Age, yrs/Sex	CMV Status (D/R)	Reason for Transplant	Time Until GBS	Maintenance Immunotherapy	IVIG	TPE	Max/Final Grade∗
Visser et al. (6)	62/M	+/+	NS	4.5 months	Cyclosporine, azathioprine, and steroids	Yes	Yes	G4/G1
Visser et al. (6)	61/M	+/−	Rheumatic	4 months	Cyclosporine, azathioprine, and steroids	Yes	Yes	G4/G2
Baldwin et al. (7)	62/M	+/−	DCM	5 days	Cyclosporine and steroids	Yes	Yes	G5/G2
El-Sabrout et al. (8)	44/M	NS	NS	14 yrs	Cyclosporine, mycophenolate mofetil, and steroids	No	Yes	G4/G1
Hodowanec et al. (9)	40/M	+/−	ICM	7 months	Cyclosporine	No	Yes	G5/G1
Steger et al. (10)	50/M	+/−	NICM	2 yrs	NS	Yes	Yes	G5/G3
Present study	56/M	−/−	ICM	21 months	Mycophenolate, mofetil, and tacrolimus	Yes	No	G2/G1

The clinical characteristics of all 7 reported cases of GBS after CMV in patients with heart transplants are summarized.

CMV = cytomegalovirus; D = donor; DCM = dilated cardiomyopathy; GBS = Guillain-Barré Syndrome; ICM = ischemic cardiomyopathy; IVIG = intravenous immunoglobulin; NICM = nonischemic cardiomyopathy; NS = not specified; R = recipient; TPE = total plasma exchange.

∗ Graded using the GBS syndrome disability (12).

IVIG monotherapy was chosen as his GBS was not severe and it avoids the risks of central venous access and further immunosuppression. All prior patients had more severe GBS and received total plasma exchange. Of the 6 prior cases, 4 were also treated with IVIG. All patients had at least partial recovery from their GBS (7, 8, 9, 10, 11).

## Conclusions

CMV infection is a common infectious complication following cardiac transplantation and can occur early or late post-transplant. GBS is a potentially lethal complication of CMV infection in this patient population. Early recognition and initiation of disease modifying therapy is crucial to prevent complications.

## Funding Support and Author Disclosures

Dr. Mitter is on the advisory board for Pfizer and Alnylam. All other authors have reported that they have no relationships relevant to the contents of this paper to disclose.
